# Slow Relaxation of the Magnetization in Anilato-Based Dy(III) 2D Lattices

**DOI:** 10.3390/molecules26041190

**Published:** 2021-02-23

**Authors:** Samia Benmansour, Antonio Hernández-Paredes, María Bayona-Andrés, Carlos J. Gómez-García

**Affiliations:** Departamento de Química Inorgánica, ICMol, Universidad de Valencia, C/Catedrático José Beltrán 2, 46980 Valencia, Spain; antonio.hernandez-paredes@uv.es (A.H.-P.); mbayonaandres@hotmail.com (M.B.-A.)

**Keywords:** SMM, SIM, anilato ligands, Dy(III), FI-SIM, honeycomb structure, layered materials

## Abstract

The search for two- and three-dimensional materials with slow relaxation of the magnetization (single-ion magnets, SIM and single-molecule magnets, SMM) has become a very active area in recent years. Here we show how it is possible to prepare two-dimensional SIMs by combining Dy(III) with two different anilato-type ligands (dianions of the 3,6-disubstituted-2,5-dihydroxy-1,4-benzoquinone: C_6_O_4_X_2_^2−^, with X = H and Cl) in dimethyl sulfoxide (dmso). The two compounds prepared, formulated as: [Dy_2_(C_6_O_4_H_2_)_3_(dmso)_2_(H_2_O)_2_]·2dmso·18H_2_O (**1**) and [Dy_2_(C_6_O_4_Cl_2_)_3_(dmso)_4_]·2dmso·2H_2_O (**2**) show distorted hexagonal honeycomb layers with the solvent molecules (dmso and H_2_O) located in the interlayer space and in the hexagonal channels that run perpendicular to the layers. The magnetic measurements of compounds **1**, **2** and [Dy_2_(C_6_O_4_(CN)Cl)_3_(dmso)_6_] (**3**), a recently reported related compound, show that the three compounds present slow relaxation of the magnetization. In compound **1** the SIM behaviour does not need the application of a DC field whereas **2** and **3** are field-induced SIM (FI-SIM) since they show slow relaxation of the magnetization when a DC field is applied. We discuss the differences observed in the crystal structures and magnetic properties based on the X group of the anilato ligands (H, Cl and Cl/CN) in **1**–**3** and in the recently reported derivative [Dy_2_(C_6_O_4_Br_2_)_3_(dmso)_4_]·2dmso·2H_2_O (**4**) with X = Br, that is also a FI-SIM.

## 1. Introduction

The last years have witnessed an increasing interest in the search for magnetic materials showing slow relaxation of the magnetization of molecular origin (single-molecule magnets, SMM) [1,2] or due to a single ion (single-ion magnets, SIM) [3,4]. The presence of slow relaxation of the magnetization and hysteresis at low temperature in SMMs is due to the presence of an energy barrier, U_eff_, for the reversal of the magnetization. This energy barrier depends on the spin ground state of the complexes and on the easy-axis magnetic anisotropy and, accordingly, the search of novel SMMs (and SIMs) with higher blocking temperatures and higher energy barriers relies on the combination of anisotropic transition metals and lanthanoids ions (Ln), with low symmetry ligands [5,6,7,8].

Although most of the SMMs and SIMs are discrete metallic complexes with different nuclearities, in recent years, some one-dimensional [9,10,11,12,13,14,15,16,17,18,19,20,21], a few two-dimensional [22,23,24,25,26,27,28,29,30,31,32,33,34] and very few three-dimensional polymers [13,34,35,36] with SMM behaviour have also been prepared.

Most of these coordination polymers are formed by Mn(II)/Mn(III) clusters of different nuclearities with SMM behaviour, linked by long bridging ligands that isolate them from the magnetic point of view [9,10,11,12,13,22,23,24,25,29]. There are also a few coordination polymers containing 3d/4f clusters [14,15,17,26,27,28] or lanthanoid ions [20,35], including one- [16,18,19,21], two- [30,31,32,33,34] and three-dimensional [34,35,36] Dy(III) coordination polymers.

Furthermore, the bridging ligands that provide good isolation from the magnetic point of view may also give rise to additional properties as photo-modulation [21], white light emission [19], photoluminescence [20,35] or even novel magnetic states [29].

Besides the search of SMMs and SIMs, lanthanoids have also been used to prepare coordination polymers and metal-organic frameworks showing interesting properties such as solvent and gas exchange or luminescence [37,38,39].

**Scheme 1:**
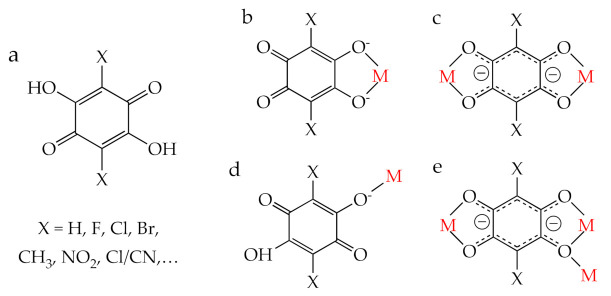
(**a**) The 3,6-disubstituted anilato derivatives (H_2_C_6_O_4_X_2_). (**b**–**e**) Some of the coordination modes of the anilato ligands: (**b**) bidentate (*1k^2^O,O′*), (**c**) bis-bidentate (*1k^2^O,O′;2k^2^O″,O‴*), (**d**) monodentate (*1kO*) and (**e**) bis-bidentate-monodentate (*1k^2^O,O′;2k^2^O″,O‴;3kO″*).

In recent years, anilato-derivative ligands (3,6-disubstituted of the 2,5-dihydroxy-1,4-benzoquinone dianion = C_6_O_4_X_2_^2−^, Scheme 1a) have been combined with Ln(III) ions to prepare coordination polymers with interesting optical [40,41,42,43] or magnetic properties, including SMMs [33,44,45,46].

Anilato ligands are very appropriate to construct these materials since: (i) they can act with different coordination modes, such as: bidentate (*1k^2^O,O′*), bis-bidentate (*1k^2^O,O’;2k^2^O″,O‴*), monodentate (*1kO*), monodentate-bidentate (*1kO;2k^2^O′,O″*) or other complex modes as (*1k^2^O,O′;2k^2^O″,O‴;3kO″*) (Scheme 1b–e) [47,48], (ii) they can connect two different metal atoms (Scheme 1c), giving rise to coordination oligomers and polymers [47], (iii) although they promote tuneable (antiferro)magnetic coupling between transition metal ions [49,50], this coupling is very weak when connecting Ln(III) ions (due to the internal character of the 4f orbitals), providing, thus, a good magnetic isolation, needed to avoid the fast relaxation of the magnetization in order to obtain SMMs and SIMs and (iv) they are equivalent to the well-known oxalato ligand (C_2_O_4_^2−^) from the topological point of view and they can form similar discrete tris(anilato)metalato complexes [51,52] and extended one-, two- and three-dimensional lattices although with larger channels and cavities [47,48,53,54,55].

In most of these coordination polymers, the Ln(III) ions are surrounded by three bidentate anilato ligands and complete their octa- or nona-coordination with two or three solvent molecules with a high coordination capacity towards Ln(III) ions [56,57] such as water, dimethyl sulfoxide (dmso), dimethylformamide (dmf), dimetylacetamide (dma), etc. [58,59,60].

Here we present the synthesis and structure of two novel coordination polymers prepared with Dy(III), dmso and two different anilato ligands: (C_6_O_4_X_2_)^2−^, with X = H (2,5-dihydroxy-1,4-benzoquinone = dhbq^2−^) and X = Cl (chloranilato). These compounds, formulated as [Dy_2_(C_6_O_4_H_2_)_3_(dmso)_2_(H_2_O)_2_]·2dmso·18H_2_O (**1**) and [Dy_2_(C_6_O_4_Cl_2_)_3_(dmso)_4_]·2dmso·2H_2_O (**2**), crystallize as hexagonal honeycomb layers with dmso and H_2_O molecules occupying the interlayer space and the hexagonal channels that run perpendicular to the layers. We also present the single-ion magnet behaviour of compounds **1** and **2** and of the closely related compound prepared with the ligand chlorocyananilato (X = Cl/CN): [Dy_2_(C_6_O_4_(CN)Cl)_3_(dmso)_6_] (**3**), whose crystal structure has been recently reported by Mercuri et al. [61]. Finally, we present a comparative structural and magnetic study of compounds **1**–**3** and of a forth closely related compound: [Dy_2_(C_6_O_4_Br_2_)_3_(dmso)_4_]·2dmso·2H_2_O (**4**), also prepared with Dy(III), dmso and an anilato ligand (bromanilato, X = Br), whose structure and magnetic properties have been recently reported by some of us [33,44].

## 2. Results

### 2.1. Synthesis

The synthesis of single crystals of the two compounds has been performed using a layering method with Dy(CH_3_COO)_3_·4H_2_O, for **1** and Dy(NO_3_)_3_·5H_2_O, for **2** as precursor salts, with the corresponding anilato acids and dmso as solvent. Surprisingly, when Dy(NO_3_)_3_·5H_2_O is used instead of Dy(CH_3_COO)_3_·4H_2_O and/or if NEt_3_ is not added to the solution, no single crystals of compound **1** could be obtained. A possible reason may be the lower acidity of H_2_dhbq (X = H) compared with the chloranilic (X = Cl) acid. Compound **3** was synthesized as compound **2** but using the precursor salt KH(C_6_O_4_(CN)Cl).

### 2.2. X-ray Crystal Structure

#### 2.2.1. Structure of [Dy_2_(C_6_O_4_H_2_)_3_(dmso)_2_(H_2_O)_2_]·2dmso·18H_2_O (**1**)

**Figure 1:**
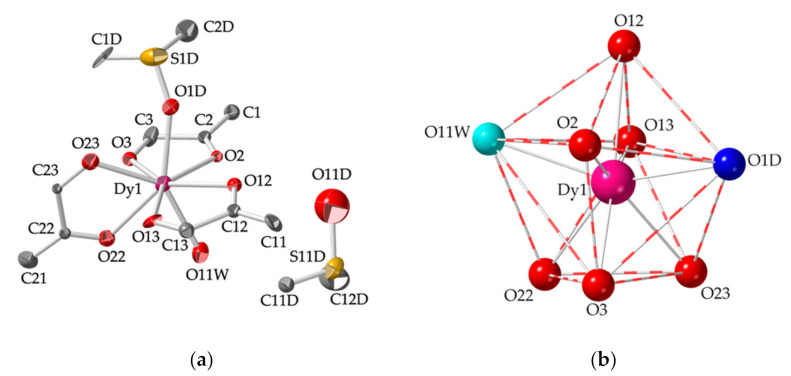
(**a**) Asymmetric unit of compound **1** with the labelling scheme (ellipsoids drawn at 50% probability). H atoms and the crystallization water molecule have been omitted for clarity; (**b**) Coordination environment of the Dy(III) ion in compound **1**. The O atom of the coordinated dmso and water molecules are drawn in dark and light blue, respectively.

The single crystal X-ray analysis shows that compound **1** crystallizes in the monoclinic *C*2*/c* space group. The asymmetric unit contains one Dy(III) ion, three halves dhbq^2−^ ligands, one coordinated dmso molecule, one coordinated water molecule, one crystallization dmso molecule and nine disordered crystallization H_2_O molecules (Figure 1a), giving rise to the formula: [Dy_2_(C_6_O_4_H_2_)_3_(dmso)_2_(H_2_O)_2_]·2dmso·18H_2_O.

The Dy(III) atoms are octa-coordinated by six oxygen atoms from three chelating chloranilato ligands (O2, O3, O12, O13, O22 and O23) and two oxygen atoms from a coordinated dmso molecule (O1D) and from a coordinated water molecule (O11W) with a triangular dodecahedron (TDD-8) coordination geometry [62], as shown by continuous SHAPE measurement analysis (Table S1, Supporting Information) [63,64,65]. The two O atoms from the coordinated dmso and H_2_O molecules are located in *trans* disposition (Figure 1b). The Dy-O bond lengths (Table 1) are similar to those of compound **2** (see below) and of the other Dy-anilato compounds with coordination number eight [44,46]. The Dy-O_dmso_ and Dy-O_water_ bond distances are shorter than the Dy–O_anilato_ ones (Table 1), due to the formation of a five membered chelate ring between the Dy centre and the anilato ligand.

**Figure 2:**
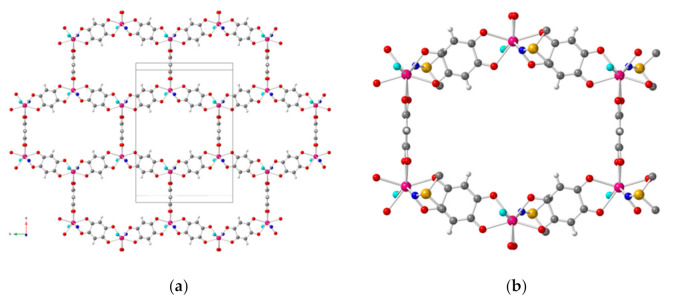
(**a**) Top view of one distorted honeycomb (3,6)-gon layer in **1** (Only the O atom of the coordinated dmso molecules is shown, for clarity); (**b**) View of one hexagonal ring in compound **1**. Colour code: Dy = pink, C = grey, S = yellow, H = white, O_anilato_ = red, O_water_ = light blue, O_dmso_ = dark blue. H atoms (except for the dhbq^2−^ ligands) and crystallization dmso molecules have been omitted for clarity.

The three coordinated bis-bidentate dhbq^2−^ ligands connect each Dy(III) ion with its three neighbours (with Dy⋯Dy distances through the dbbq^2−^ bridges of 8.55 and 8.58 Å), giving rise to neutral layers, parallel to the *ab* plane, formulated as [Dy_2_(C_6_O_4_H_2_)_3_(dmso)_2_(H_2_O)_2_] and formed by distorted hexagons with a 3,6-gon honeycomb topology (Figure 2a). Each hexagon contains six Dy(III) ions located in the vertices and six dhbq^2−^ ligands forming the sides of the hexagons (Figure 2b). Four anilato rings are oriented parallel to the layer (face-on, *FO*) and the other two are oriented nearly perpendicular to the layer (edge-on, *EO*, Figure 2b), giving rise to the so-called phase IIb [55,58].

Although not very common, distorted hexagonal lattices have already been found in other Ln-anilato compounds with different anilato ligands, solvents and Ln(III) ions [40,41,44,55,58,59,60,66,67]. In contrast, phase IIb is rather unusual and has only been observed in a few examples of Ln(III) with different anilato ligands (X = Cl, Br, Cl/CN and *t*-Bu) [40,41,44,48,49,50,51,52,53,54,55,56,57,58,59,60,67], although it had never been observed with dhbq^2−^ [55]. Therefore, compound **1** is the first example of phase IIb with the ligand dhbq^2−^.

Two consequences of the distortion of the hexagons are: (i) the deviation from 120° of the Dy–Dy–Dy angles (144.66°, 107.61° and 107.61°) and (ii) the different Dy-Dy distances along the diagonals of the hexagons (17.761, 18.419 and 18.419 Å).

**Figure 3:**
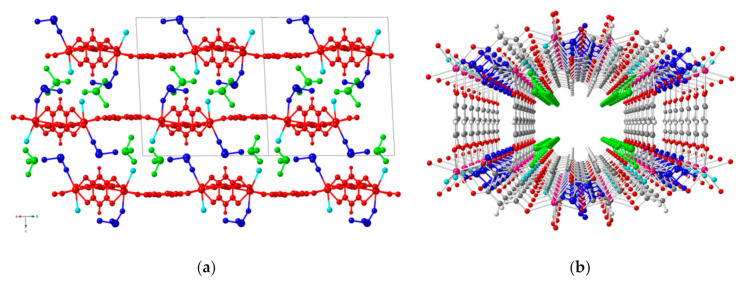
(**a**) Side view of three consecutive layers in compound **1** (in red) showing the coordinated dmso and water molecules (dark and light blue, respectively) and the crystallization dmso molecules (green); (**b**) Perspective view of two rectangular and one hexagonal channels in **1** along the *c* direction showing the crystallization dmso (in green) located in the hexagonal channels.

The hexagonal layers are almost planar (Figure 3a), as confirmed by the sum of the three Dy–Dy–Dy angles of 359.88°, very close to 360°, the expected value for a planar hexagon. The coordinated dmso and water molecules (dark and light blue, respectively, in Figure 3a) are oriented almost orthogonal to the layers pointing towards the interlaminar space, whereas the crystallization dmso molecules (green in Figure 3a) occupy the interlayer space.

The layers are packed in a slightly alternating way along the *c* direction (perpendicular to the layers), giving rise to the formation of small empty rectangular channels together with large hexagonal channels along the *c* direction (Figure 3b). The large hexagonal channels contain the crystallization dmso molecules and the disordered crystallization H_2_O molecules. The MASK analysis of these hexagonal channels shows that they represent ca. 39% of the unit cell volume and contain 18 H_2_O molecules per hexagon (i.e., [Dy_2_(C_6_O_4_H_2_)_3_(dmso)_2_(H_2_O)_2_]), although the low quality of the crystal precludes the exact determination of the location of the water molecules.

#### 2.2.2. Structure of [Dy_2_(C_6_O_4_Cl_2_)_3_(dmso)_4_]·2dmso·2H_2_O (**2**)

**Figure 4:**
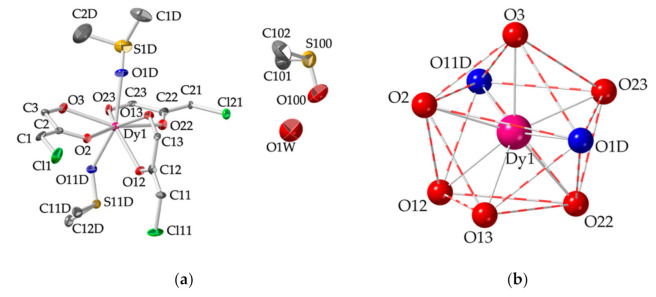
(**a**) Asymmetric unit of compound **2** with the labelling scheme (ellipsoids drawn at 50% probability). H atoms have been omitted for clarity; (**b**) Coordination environment of the Dy(III) ion in compound **2**. O atoms of the coordinated dmso are drawn in dark blue.

The single crystal X-ray analysis shows that compound **2** crystallizes in the triclinic *P*-1 space group. The asymmetric unit contains one Dy(III) ion, three halves chloranilato ligands, two coordinated dmso molecules and one dmso and one crystallization H_2_O molecules (Figure 4a), resulting in the formula: [Dy_2_(C_6_O_4_Cl_2_)_3_(dmso)_4_]·2dmso·2H_2_O.

The Dy(III) atoms are octa-coordinated by six oxygen atoms from three chelating chloranilato ligands (O2, O3, O12, O13, O22 and O23) and two oxygen atoms (O1D and O11D) from two coordinated dmso molecules. Continuous SHAPE measurement analysis [64,65] indicates that the coordination geometry around the Dy(III) ion is also a triangular dodecahedron (TDD-8, Table S1, Supporting Information) [62] with the two O atoms from the dmso molecules in *trans* disposition (Figure 4b). The Dy-O bond lengths (Table 1) are similar to those of compound **1** and of the other Dy-anilato compounds with the same coordination number [44,46]. As observed in compound **1**, the Dy-O_dmso_ bond distances are shorter than the Dy–O_anilato_ ones, due to the chelating coordination mode of the chloranilato ligands (Table 1).

**Figure 5:**
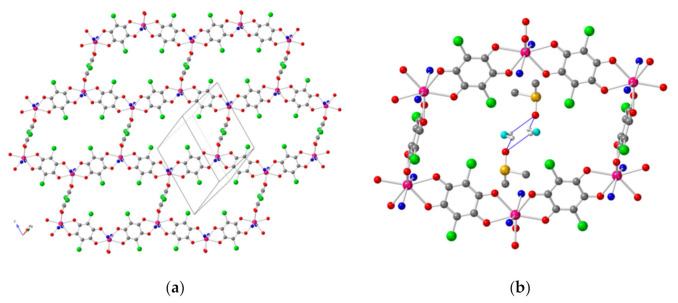
(**a**) Top view of one distorted honeycomb (3,6)-gon layer in **2**; (**b**) View of one hexagonal ring in compound **2**. Thin blue lines represent the H-bonds between the water molecules and the O atoms of the crystallization dmso molecules. Colour code: Dy = pink, C = grey, S = yellow, Cl = green, O_anilato_ = red, O_water_ = light blue, O_dmso_ = dark blue. Only the O atom of the coordinated dmso molecules is shown. H atoms and crystallization dmso molecules (only in (**a**)) have been omitted for clarity.

The three coordinated bis-bidentate chloranilato ligands connect each Dy(III) with three other Dy(III) ions (with Dy⋯Dy distances through the chloranilato bridges of 8.65, 8.69 and 8.63 Å), giving rise to neutral distorted hexagonal layers with the classical 3,6-gon honeycomb topology formulated as [Dy_2_(C_6_O_4_Cl_2_)_3_(dmso)_4_] (Figure 5a). The hexagons are formed by six Dy(III) ions and six chloranilato ligands located in the vertices and sides of the hexagons, respectively. Four of the six anilato rings are parallel to the layer (face-on orientation, *FO*) whereas the other two are perpendicular to the layers (edge-on orientation, *EO*, Figure 5b). This orientation of the anilato rings gives rise to the so-called phase IIb [55,58]. This phase IIb with chloranilato has only been reported for Gd(III) and Eu(III) with H_2_O as solvent [58] and for Er(III) with H_2_O, dimethylformamide (dmf) and dmso [60], but never with Dy(III). Therefore, compound **2** is the first example of phase IIb with Dy(III) and chloranilato.

As in compound **1**, the distortion of the hexagons gives rise to different Dy-Dy-Dy angles (141.78°, 122.36° and 94.87°) and Dy-Dy distances along the diagonals of the hexagons (14.115, 16.606 and 20.247 Å).

**Figure 6:**
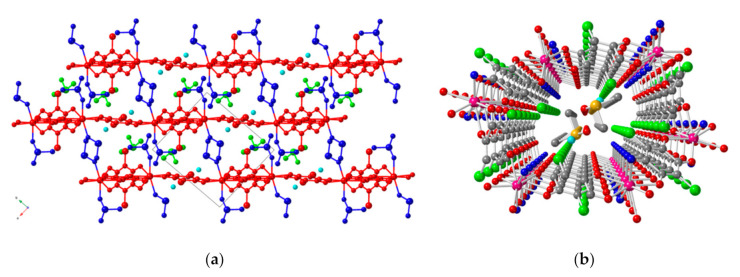
(**a**) Side view of three consecutive layers in **2** (in red) showing the coordinated dmso molecules (dark blue), the crystallization dmso molecules (green) and the crystallization water molecules (ligh blue); (**b**) Perspective view of one hexagonal channel in **2** along the *b* direction showing the crystallization dmso and water molecules located in the channels. Colour code in **b**: Dy = pink, C = grey, S = yellow, Cl = green, O_anilato_ = red, O_water_ = light blue, O_dmso_ = dark blue. H atoms have been omitted for clarity.

The hexagonal layers are almost planar (the sum of the three Dy-Dy-Dy angles is 358.92°, Figure 6a). The coordinated dmso molecules are oriented almost orthogonal to the layers pointing towards the interlaminar space (dark blue in Figure 6a). The crystallization dmso molecules are located in the interlayer space (green in Figure 6a) whereas the water molecules (light blue in Figure 6a) are located very close to the hexagonal plane, in the hexagonal cavities, forming H-bonds with the O atoms of the crystallization dmso molecules (Figure 5b) with O⋯O distances of 2.865 and 2.963 Å [68].

A final interesting aspect of the structure of compound **2** is the formation of an eclipsed packing of the layers along the *b* direction. This packing gives rise to hexagonal channels along the *b* direction that contain the crystallization dmso and water molecules (Figure 6b).

## 3. Discussion

### 3.1. Structural Comparison of Compounds [Dy_2_(C_6_O_4_H_2_)_3_(dmso)_2_(H_2_O)_2_]·2dmso·18H_2_O (***1***), [Dy_2_(C_6_O_4_Cl_2_)_3_(dmso)_4_]·2dmso·2H_2_O (***2***), [Dy_2_(C_6_O_4_(CN)Cl)_3_(dmso)_6_] (***3***) and [Dy_2_(C_6_O_4_Br_2_)_3_(dmso)_4_]·2dmso·2H_2_O (***4***)

Besides compounds **1** and **2**, there are two, very recently reported compounds, also prepared with dmso, containing Dy(III) and chlorocyananilato (X = Cl/CN) or bromanilato (X = Br) ligands with similar 3,6-gon lattices. These compounds are: [Dy_2_(C_6_O_4_(CN)Cl)_3_(dmso)_6_] (**3**) [61] and [Dy_2_(C_6_O_4_Br_2_)_3_(dmso)_4_]·2dmso·2H_2_O (**4**) [44]. Compounds **1**–**4** show SIM behaviour (see below), although it has only been very recently reported in compound **4** [33]. Besides the X group in the anilato ligand and the number of crystallization solvent molecules, the main difference among compounds **1**–**4** is the number and type of coordinated solvent molecules: one H_2_O and one dmso in **1**, two dmso molecules in **2** and **4** and three dmso molecules in **3**. Therefore, the main difference is the nona-coordination of the Dy(III) ions in **3** vs. their octa-coordination in **1**, **2** and **4**.

Interestingly, a similar change in the coordination number (from nine to eight), has recently been observed in the related series [Ln_2_(C_6_O_4_Br_2_)_3_(dmso)_n_]·2dmso·mH_2_O with n/m = 6/0 for Ln = La-Gd and n/m = 4/2 for Ln = Tb–Tm [44]. Nevertheless, in this series the change in the coordination number is easy to explain based on the size of the Ln(III) ions. Thus, the smaller Ln(III) ions (from Tb to Tm) are octa-coordinated whereas the larger ones (from La to Gd) are nona-coordinated. Another similar change in the coordination number has been observed in two closely related series formulated as [Er_2_(C_6_O_4_Cl_2_)_3_(L′)_n_]·G and [Er_2_(C_6_O_4_Br_2_)_3_(L′)_6_]·G, prepared with the same lanthanoid ion (Er) and chloranilato (X = Cl) or bromanilato (X = Br). In the chloranilato series the coordination number is eight (n = 4) when the solvent molecule is large (L′ = formamide = fma, dimethylformamide = dmf or hexamethylphosphormamide = hmpa) whereas it is nine (n = 6) when the coordinating solvent molecules are small (L′ = H_2_O, dmso or dimethylacetamide = dma) [60]. In the bromanilato series the coordination number is also eight for the larger solvent molecule (L′ = dmso) but it is nine when the solvent molecules are smaller (L′ = H_2_O and dmf) [59]. In these two Er(III)-containing series, the change in the coordination number is attributed to a change in the size and/or steric hindrance of the solvent molecules.

Albeit, in compounds **1**–**4**, the change in the coordination number when passing from compounds **1**, **2** and **4** (with coordination number of eight) to compound **3** (with coordination number of nine), has to be attributed to the change in the X group (X = H, Cl, Cl/CN and Br in **1**–**4**, respectively) since the Ln(III) ion and the coordinated solvent molecules are the same (except in compound **1** where a dmso molecule has been replaced by an H_2_O molecule).

Moreover, a detailed study of compound **3**, performed by Mercuri et al. [61], shows that this compound may crystallize as two different phases when recrystallized from dmso: (i) a double square (3,8)+(3,4)-2D lattice formulated as [Dy_2_(C_6_O_4_(CN)Cl)_3_(dmso)_6_]·7H_2_O (**3**′) and (ii) a rectangular brick-wall (3,6)-2D lattice formulated as [Dy_2_(C_6_O_4_(CN)Cl)_3_(dmso)_6_] (**3**). Both phases contain nona-coordinated Dy(III) ions with a capped square antiprismatic coordination geometry (CSAPR-9) formed by three bidentate chlorocyananilato ligands and three coordinated dmso molecules. The only difference is the distortion of the CSAPR-9 geometry, which is more important in compound **3**, as clearly shown by the continuous SHAPE measurement analysis, with values of 0.114 and 0.500 for the CSAPR-9 geometry in **3**′ and **3**, respectively [64,65]. From the synthetic point of view, the only differences are the concentration of the recrystallization solution (0.33 mg/mL in **3** vs. 1 mg/mL in **3**′) and the recrystallization time (2 weeks in **3** vs. 2–5 days in **3**′), suggesting that **3**′ is the kinetic phase whereas **3** is the thermodynamic one. In our case, we obtained compound **3** after two weeks (see Experimental Section) and, as expected, we have obtained the thermodynamic phase (compound **3**). Although the synthesis of **3** and **3**′ indicates that the synthetic conditions are a key factor in determining the final structure, it does not explain why the Dy(III) ions in compounds **3** and **3**′ are nona-coordinated but they are octa-coordinated in compounds **1**, **2** and **4**.

In order to answer this question, we have, therefore, to focus on the anilato ligands. In compounds **3** and **3**′ the Cl and CN groups exert a strong electron withdrawing effect on the anilato ring that reduces the electron density on the coordinating O atoms, resulting in weaker (and longer) Dy-O_anilato_ bonds. These longer Dy-O bonds allow the coordination of up to three dmso molecules. In contrast, in compounds **1**, **2** and **4**, the electron withdrawing effect is smaller, resulting in stronger (and shorter) Dy-O_anilato_ bonds that reduce the available space around the Dy(III) ions and preclude the coordination of a third dmso molecule. This assumption is confirmed by the much shorter average Dy-O_anilato_ bond distances in compounds **1**, **2** and **4** (2.365, 2.384 and 2.384 Å, respectively) than in compounds **3** and **3′** (2.457 and 2.453 Å, respectively). A similar effect was observed in the chloranilato and bromanilato 2D honeycomb heterometallic lattices with Mn(II) and Cr(III): [MnCr(C_6_O_4_Cl_2_)_3_(H_2_O)]^−^ and [MnCr(C_6_O_4_Br_2_)_3_]^−^, where the more electron withdrawing X group (Cl) leads to a higher coordination number (seven) and the less electron withdrawing X group (Br) results in a lower coordination number (six) [49].

**Figure 7:**
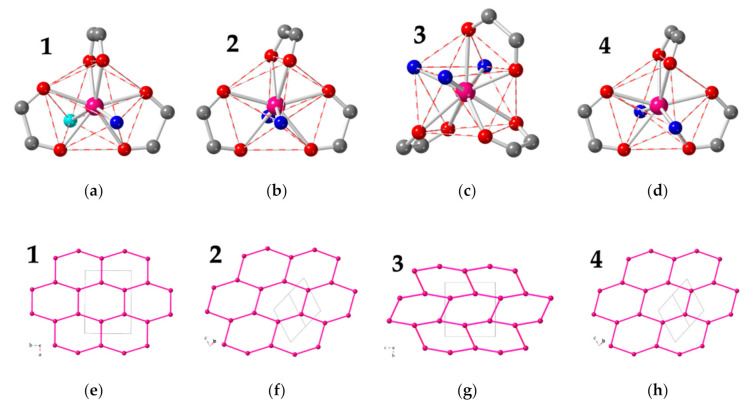
(**a**–**d**) Coordination polyhedra of compounds **1**–**4**. Colour code: Dy = pink, C = grey, O_anilato_ = red, O_water_ = light blue, O_dmso_ = dark blue. (**e**–**h**) Schematic view of the layers in compounds **1**–**4**. Only the Dy atoms are shown for clarity. The pink lines represent the anilato bridges.

The key role in the final structure of the coordination geometry around the Dy(III) ion observed in **3** and **3′**, is further confirmed in compounds **1**, **2** and **4**. Thus, compounds **1**, **2** and **4** present similar distorted hexagonal honeycomb structures whereas compound **3** presents a brick-wall structure with almost rectangular cavities. These differences in the shape of the cavities are directly related to the orientation of the anilato ligands in the coordination polyhedra of the Dy(III) ions in compounds **1**–**4** (Figure 7a–d). Thus, in compounds **1**, **2** and **4** the spatial orientation of the anilato ligands is the same and, consequently, they present similar distorted hexagonal layers. Furthermore, compounds **2** (X = Cl) and **4** (X = Br) are isostructural [44]. In contrast, compound **3** shows a different coordination number and geometry (although with a similar spatial disposition of the three anilato ligands) and, accordingly, shows a much more distorted structure although with the same 3,6-gon topology (Figure 7e–h).

### 3.2. Magnetic Properties of Compounds ***1**–**4***

Although the DC magnetic properties of compound **3** have already been reported [61], we include them only for comparative purposes. In contrast, AC measurements of compound **3** have not been reported yet and, therefore, here we will show a detailed study of the AC magnetic properties of this compound, as well as those of compounds **1** and **2**.

The DC magnetic measurements of compounds **1**–**3** show very similar behaviours for the three compounds. The product of the molar magnetic susceptibility per two Dy(III) ions times the temperature (χ_m_T) is around 28.5 cm^3^ K mol^−1^ at room temperature for the three compounds and shows a smooth decrease when the samples are cooled to reach values of ca. 21.0, 23.5 and 22.5 cm^3^ K mol^−1^ at 2 K for **1**−**3**, respectively (Figure S1a, Supporting Information). The room temperature value is close to the expected one for two isolated Dy(III) ions with a ^6^H_15/2_ ground multiplet and g = 4/3 and the thermal behaviour is also the expected one for magnetically isolated Dy(III) ions and is attributed to the depopulation of the excited sublevels appearing due to the ligand field [69]. The magnetic isolation of the Dy(III) ions is not surprising and has been observed in other compounds with Ln(III) ions connected through bis-bidentate anilato ligands [40,44,59,60,70,71].

The isothermal magnetization at 2 K also shows the expected behaviour for isolated Dy(III) ions with a rapid increase at low fields and a smooth and linear increase at high fields with a value of ca. 6 μ_B_ per Dy(III) ion at high fields (Figure S1b, Supporting Information) and shows saturation values close to those observed in other Dy(III) compounds with isolated Dy(III) ions [72,73].

**Figure 8:**
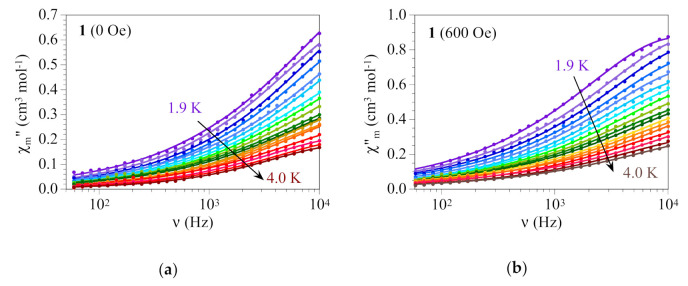
(**a**) Frequency dependence of χ″_m_ for compound **1** with H_dc_ = 0 Oe in the temperature range 1.9–4.0 K; (**b**) Frequency dependence of χ″_m_ for compound **1** with H_dc_ = 600 Oe in the temperature range 1.9–4.0 K. Solid lines are the best fit to the Debye model.

The AC magnetic measurements for compound **1** show slow relaxation of the magnetization at low temperatures with and without an applied DC field. Thus, the frequency dependence of the in–phase (χ′_m_) and out–of–phase (χ″_m_) signals for compound **1** with no DC field shows a decrease in χ′_m_ (Figure S2a, Supporting Information) and an increase in χ″_m_ at high frequencies (Figure 8a) with no maximum below 10 kHz, indicative of slow relaxation of the magnetization at low temperatures. Although the inflexion point in χ′_m_ and the maximum in χ″_m_ are not reached at 10 kHz, we can observe that the maximum slope in χ″_m_ is located near 2–3 kHz and, accordingly, we can assume that the maximum in χ″_m_ must be located slightly above 10 kHz, although the exact position cannot be determined with our set of data. Despite this uncertainty, we have fitted both signals, χ′_m_ and χ″_m_, with the Debye model (solid lines in Figure S2a and Figure 8a) to obtain the corresponding relaxation times in the temperature range 1.9–4.0 K.

When a DC magnetic field is applied at 1.9 K, the χ″_m_ signal increases with increasing the DC field and reaches a maximum at around 600 Oe at high frequencies (that shifts to higher fields for lower frequencies, Figure S3, Supporting Information). The study of the frequency dependence at 1.9 K for different DC fields (Figure S4, Supporting Information) shows that the intensity of χ″_m_ increases as the DC field increases and reaches a maximum value at ca. 600 Oe. For all DC fields the maximum of χ″_m_ appears above 10 kHz and, therefore, the fit of the frequency dependence of χ″_m_ to a Debye model is not very precise. Nevertheless, we observe that the relaxation time (τ) presents a maximum near 600 Oe and that the field dependence of τ follows the expected variation with n = 4 for Kramers ions (Figure S5, Supporting Information) [74]:

(1) $$\tau^{-1}={\mathrm{ATH}}^{n}+\frac{B_{1}}{1+B_{2}H^{2}}+D$$

Accordingly, we have performed AC measurements at different temperatures and frequencies with a DC field of 600 Oe. These measurements show again frequency-dependent χ′_m_ and χ″_m_ signals (Figure S2b and Figure 8b) very similar behaviour to those performed with no applied DC field that can also be fitted to the Debye model (solid lines in Figure S2b and Figure 8b).

The Cole-Cole plots (χ″_m_ vs. χ′_m_, Figure S6, Supporting Information) show a fragment of a semicircle, confirming the presence of slow relaxation of the magnetization.

**Figure 9:**
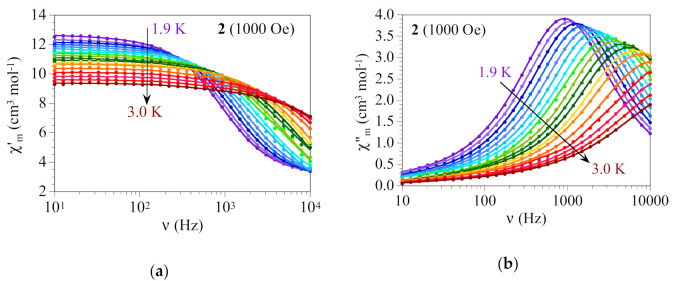
(**a**) Frequency dependence of χ′_m_ for compound **2** with H_dc_ = 1000 Oe in the temperature range 1.9–3.0 K; (**b**) Frequency dependence of χ″_m_ for compound **2** with H_dc_ = 1000 Oe in the temperature range 1.9–3.0 K. Solid lines are the best fit to the Debye model.

For compound **2** the AC measurements with no applied DC field do not show any AC signal, probably due to the presence of a fast quantum tunnelling process for H_DC_ = 0 Oe. Albeit, when a DC field is applied, we can observe the appearing of χ′_m_ and χ″_m_ signals at low temperatures with a maximum χ″_m_ signal for a DC field of ca. 1000 Oe. (Figure S7, Supporting Information). The frequency dependence of χ″_m_ at 1.9 K with different applied DC fields for compound **2** shows a clear maximum in χ″_m_ whose intensity increases as the DC field increases and reaches a maximum intensity at around 900–1000 Oe (Figure S8, Supporting Information). The relaxation times obtained with the fit of χ″_m_ to de Debye model show a maximum at ca. 1000 Oe (0.1 T, Figure S9, Supporting Information) and follow the expected field dependence (Equation (1)). Accordingly, we have performed AC measurements at different frequencies and temperatures with an applied DC field of 1000 Oe (Figure 9). These measurements show an inflexion in χ′_m_ that shifts to higher frequencies as the temperature increases (Figure 9a) and a maximum in χ″_m_ that also shifts to higher frequencies with increasing temperature (Figure 9b) that can be well reproduced with the Debye model (solid lines in Figure 9). The Cole-Cole plot of compound **2** with H_DC_ = 1000 Oe also shows a semi-elliptical curve although now we can see a much larger fragment at low temperatures (Figure S10, Supporting Information).

**Figure 10:**
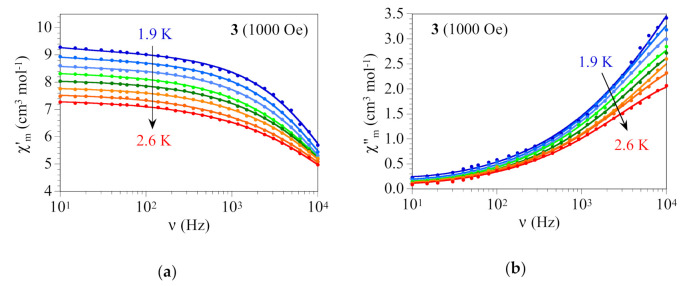
Frequency dependence of χ′_m_ (**a**) and χ″_m_ (**b**) for compound **3** with H_dc_ = 1000 Oe in the Table 1. 9–2.6 K. Solid lines are the best fit to the Debye model.

Finally, compound **3** also shows slow relaxation of the magnetization although, as can be seen in the field dependence of χ″_m_ for compound **3** at 1.9 K (Figure S11, Supporting Information), we need to apply a DC field to observe the SIM behaviour, probably, as in **2**, due to the presence of a fast quantum tunnelling process for H_DC_ = 0 Oe. Since the maximum in the χ″_m_ signal is observed for fields of ca. 1000 Oe, we have performed AC measurements at different frequencies and temperatures with H_DC_ = 1000 Oe. These measurements suggest the presence of an inflexion point in the frequency dependence of χ′_m_ above 10 kHz (Figure 10a). The χ″_m_ signal shows an increase at high frequencies although no maximum is observed below 10 kHz (Figure 10b). Although, as in compound **1**, we do not observe the inflexion point in χ′_m_ nor the maximum in χ″_m_, we have fitted the frequency dependence of χ′_m_ and χ″_m_ to a Debye model to obtain approximate relaxation times at very low temperatures.

A further confirmation of the presence of a relaxation process is provided by the Cole-Cole plot that shows a semicircle that can be well reproduced with the Debye model although we only observe a small fraction of the semicircle (Figure S12, Supporting Information).

**Figure 11:**
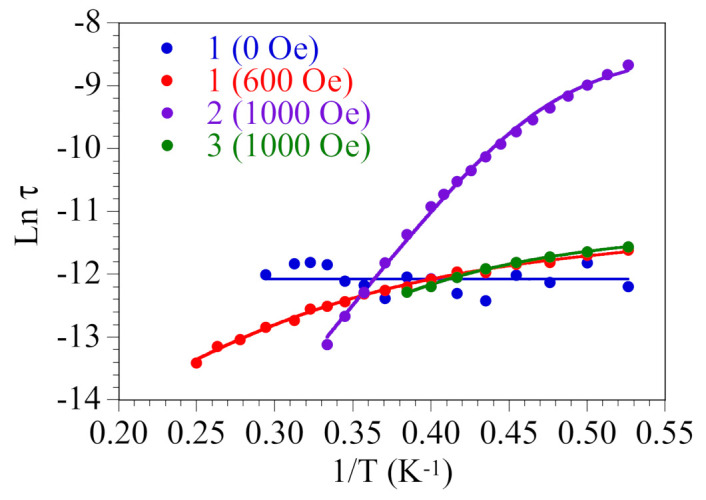
Arrhenius plot of the relaxation times for compound **1** with H_DC_ = 0 and 600 Oe, and for compounds **2** and **3** with H_DC_ = 1000 Oe. Solid lines are the best fit to the general model (Equation (2)) with different terms (see text and Table 2).

The Arrhenius plots of the relaxation times in compounds **1**–**3** (Figure 11) show an almost horizontal straight line for **1** when H_DC_ = 0 Oe and a curvature for H_DC_ = 600 Oe. In compounds **2** and **3** the relaxation times for H_DC_ = 1000 Oe also show a curvature (very slight in **3** and more pronounced in compound **2**).

In order to fit the relaxation times, we have used the general model including all the possible mechanisms: quantum tunnelling (QT, first term), direct (D, second term), Raman (R, third term) and Orbach (O, fourth term) (Equation (2)) [75]:

(2) $$\tau^{-1}=\tau_{\mathrm{QTM}}^{-1}+{\mathrm{AH}}^{2}T+{\;\mathrm{CT}}^{n}+{\;\tau }_{0}^{-1}\exp\left(\frac{-U_{\mathrm{eff}}}{k_{B}T}\right)$$

For compound **1** with H_DC_ = 0 Oe, despite the uncertainty, we observe an approximately temperature independent behaviour of the relaxation time (Figure 11), suggesting that only the quantum tunnelling mechanism is operative at very low temperatures. Accordingly, we have fitted the relaxation times considering only a quantum tunnelling term (see Table 2). In contrast, when a DC field is applied, the relaxation of the magnetization in compounds **1**–**3** follow direct and thermally activated Orbach mechanisms (Table 2) with activation energies in the range 17.1–31.5 K, similar to the activation energies observed in the very few reported examples of 2D lattices with Dy and anilato ligands: [Dy_2_(C_6_O_4_Br_2_)_3_(H_2_O)_6_]·8H_2_O (9.6 K) [33], [Dy_2_(C_6_O_4_Br_2_)_3_(dmf)_6_] (11.4 and 36 K) [33] and [Dy_0.04_Eu_1.96_(C_6_O_4_Br_2_)_3_(dmso)_6_]·2dmso (40.9 K) [45]. Of course, in compounds **1** and **3** the obtained values have to be considered with caution, given the uncertainty in the fit of the AC signals to the Debye model.

**Figure 12:**
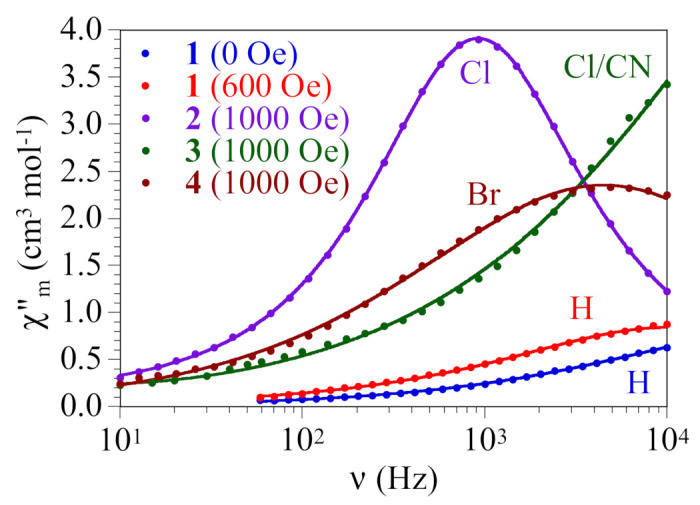
Frequency dependence of χ″_m_ for compounds **1** (X = H; H_dc_ = 0 and 600 Oe), **2** (X = Cl; H_dc_ = 1000 Oe), **3** (X = Cl/CN; H_dc_ = 1000 Oe) and **4** (X = Br; H_dc_ = 1000 Oe) at 1.9 K. Solid lines are the best fit to the Debye model.

As mentioned above, the DC magnetic properties of compounds **1** and **2** are very similar and also similar to those of compounds **3** and **4** [33,61]. The only differences are, therefore, observed in the AC magnetic properties (Figure 12).

Note that the AC magnetic properties of the X = Br derivative (compound **4**) have been recently reported by some of us [33] and, therefore, we include this compound here to compare with **1**–**3**.

The main difference between compounds **1**–**4** is the presence of slow relaxation in compound **1** with no applied DC field. This relaxation is, nevertheless, quite fast since the maximum of the frequency dependence of χ″_m_ cannot be observed below 10 kHz and, most probably, it follows a temperature-independent quantum tunnelling mechanism. A possible reason to explain this different behaviour may be the presence of a more anisotropic coordination environment of the Dy(III) ions in compound **1** (formed by three bidentate anilato ligands, one dmso and one water molecule) compared to compounds **2**–**4**, where the coordination environment only contains three bidentate anilato ligands and two (in **2** and **4**) or three (in **3**) dmso molecules (Figure 7a–d).

When a DC field is applied in compounds **1**–**3** the quantum tunnelling mechanism is cancelled and the magnetization relaxes through direct and Orbach mechanisms. Albeit, the activation energies, U_eff_, and the relaxation times, τ_0_, show some differences (Table 2). These differences may be attributed to: (i) structural effects due to changes in the coordination of the Dy(III) ions and/or (ii) electronic effects, due to changes in the donor capacity of the anilato ligands as a consequence of the different electron withdrawing capacity of the X group.

On one hand, the structural effect can be approximately quantified by the distortion from the ideal TDD-8 coordination geometry of the Dy(III) ions since they all show the same connectivity and the same disposition of the bridging ligands and solvent molecules (Figure 7). Continuous shape analysis (Table S1, Supporting Information) shows distortion values from ideal TDD-8 geometry of 0.902 in **1**, 1.208 in **2** and 1.079 in **4** [33]. On the other hand, the electronic effect can be quantified by the electronegativity of the X group of the anilato ligand (H in **1**, Cl in **2** and Br in **4**).

Although there are only three compounds to establish a correlation, we observe an almost linear dependence of both parameters (Ln τ_0_ and U_eff_) with both the distortion parameter and with the electronegativity of the X group. Thus, Ln τ_0_ decreases (blue line in Figure S13) and U_eff_ increases (blue line in Figure S14) as the distortion parameter increases. The same trend is observed for the electronegativity of the X group: Ln τ_0_ decreases (red line in Figure S13) and U_eff_ increases (red line in Figure S14) as the electronegativity increases.

Although we need to synthesize and characterize other similar examples with Dy(III) and different anilato ligands to confirm these trends, we observe that the larger the distortion of the coordination polyhedron around the Dy(III) ion, the higher U_eff_ and the lower τ_0_. On the other side, when the electron withdrawing capacity of X increases, the ligand-Dy interaction decreases, resulting in the same effect: an increase of U_eff_ and a decrease of τ_0_. These trends agree with the idea that the larger the distortion and the weaker the metal-ligand interaction, the larger the anisotropy.

## 4. Materials and Methods

### 4.1. Synthesis of Compounds ***1**–**3***

All the chemicals are commercially available and were used as received without further purification. The reactions were performed in open air. The potassium acid salt of the chlorocyananilato ligand, KH(C_6_O_4_(CN)Cl), was prepared following the method reported in the literature [76].

#### 4.1.1. Synthesis of [Dy_2_(C_6_O_4_H_2_)_3_(dmso)_2_(H_2_O)_2_]·2dmso·18H_2_O (**1**)

Dark pink prismatic single crystals of compound **1** were obtained by carefully layering, at room temperature, a solution of Dy(CH_3_COO)_3_·4H_2_O (0.01 mmol. 3.4 mg) in 2.5 mL of methanol onto a solution of 2,5-dihydroxy-1,4-benzoquinone, H_4_C_6_O_4,_ (0.01 mmol, 1.4 mg) and triethylamine (0.36 mmol, 50 µL) in 2.5 mL of dmso. The tube was sealed and allowed to stand for about 4 months. Suitable crystals for X-ray diffraction were freshly picked and covered with paratone oil in order to avoid solvent loss to be characterized by single crystal X-ray diffraction. FT-IR (ν/cm^−1^, KBr pellets): 3432 (m), 2990 (w), 2910 (w), 1522 (vs), 1374 (m), 1262 (m), 1022 (m), 955 (w), 831 (w), 712 (w), 494 (w). This compound losses very quickly in an irreversible way part of the solvent molecules when exposed to air, resulting in a loss of crystallinity that precludes the acquisition of an X-ray powder diffractogram of this compound.

#### 4.1.2. Synthesis of [Dy_2_(C_6_O_4_Cl_2_)_3_(dmso)_4_]·2dmso·2H_2_O (**2**)

Pink prismatic single crystals of **2** were obtained by carefully layering, at room temperature, a solution of chloranilic acid, H_2_C_6_O_4_Cl_2_ (0.02 mmol, 4.2 mg) in 5 mL of methanol onto a solution of Dy(NO_3_)_3_·5H_2_O (0.02 mmol. 8.77 mg) in 5 mL of dmso. The tube was sealed and allowed to stand for about 6 weeks. Suitable crystals for X-ray diffraction were freshly picked and covered with paratone oil in order to avoid solvent loss to be characterized by single crystal X-ray diffraction.

This compound was also obtained in a one-pot synthesis as a polycrystalline sample by adding, drop-wise, a solution of Dy(NO_3_)_3_·5H_2_O (0.1 mmol. 43.9 mg) in 10 mL of dmso to a solution of chloranilic acid, H_2_C_6_O_4_Cl_2_ (0.15 mmol, 31.5 mg) in 10 mL of dmso at 40 °C. After 30 min stirring at 40 °C the solution was covered and allowed to stand at room temperature. Three days later, a dark purple solid precipitated. The solution was filtered and air dried. Yield: 45%. FT-IR (ν/cm^−1^, KBr pellets): 3421 (m), 2998 (w), 2918 (w), 1495 (vs), 1381 (s), 1295 (w), 1000 (m), 959 (m), 846 (m), 712 (w), 604 (w), 579 (m), 451 (m).

Phase purity was confirmed by the X-ray powder diffractogram of compound **2** that corresponds with the simulated one from the single crystal X-ray structure (Figure S15, Supporting Information).

#### 4.1.3. Synthesis of [Dy_2_(C_6_O_4_(CN)Cl_2_)_3_(dmso)_6_] (**3**)

This compound was obtained as compound **2** but using KH(C_6_O_4_(CN)Cl) (0.15 mmol, 35.7 mg) instead of H_2_C_6_O_4_Cl_2_. After 30 min stirring at 40 °C the solution was covered and allowed to stand at room temperature. Two weeks later, a purple solid precipitated. The solution was filtered and air dried. Yield: 13%. FT-IR (ν/cm^−1^, KBr pellets): 3419 (m), 3000 (w), 2921 (w), 2213 (m), 1520 (vs), 1415 (m), 1002 (m), 958 (m), 861 (m), 713 (w), 612 (m), 482 (w), 445 (m).

The X-ray powder diffractogram of compound **3** corresponds well with the simulated one from the single crystal X-ray structure reported by Mercuri et al. [61] for compound [Dy_2_(C_6_O_4_(CN)Cl)_3_(dmso)_6_] (Figure S16), confirming the phase purity of this compound.

### 4.2. X-ray Single Crystal Structure Determination

Suitable crystals of compounds **1** and **2** were freshly picked from the mother liquor, immediately coated with paratone oil, mounted on a mylar loop and then transferred directly to the cold-nitrogen stream for data collection. X-ray data were collected at 120 K using ω scans on a Supernova diffractometer equipped with a graphite-monochromated Enhance (Mo) X-ray source (λ = 0.71073 Å). The program CrysAlisPro (Oxford Diffraction Ltd., Oxfordshire, UK) was used for unit cell determinations, data reduction, scaling and for a multi-scan absorption correction using spherical harmonics implemented in SCALE3 ABSPACK. The structures were solved in the space groups *C*2/*c* (for **1**) and *P*-1 (for **2**) determined by the ShelXT structure solution program using the Intrinsic Phasing solution method [77] and refined by least squares using version 2017/1 of XL [78]. All non-hydrogen atoms were refined anisotropically. Hydrogen atom positions were calculated geometrically and refined using the riding model. Data collection and refinement parameters are provided in Table 3.

Compound **1** shows a positional disorder in one C atom of the coordinated dmso molecule that appears on two close positions (C2D and C2D′, separated by 1.28 Å) that appear with occupancies of 0.75 and 0.25, respectively. In all the drawings only the C2D atom is drawn, since C2D′ is too far (2.125 Å) from the S atom of the dmso molecule. For compound **1**, due to severely disordered solvent molecules, the solvent contributions to the structure factors were taken into account by applying the MASK procedure in the OLEX2 program package [79]. Solvent accessible voids and total electron counts found per cell for **1** are 2033.6 Å^3^ (39%) and 692.1, respectively. This is consistent with the presence of ca. 9 H_2_O per formula unit (ca. 18 molecules per two Dy ions). Refinement details and explanations are included in the CIF file. CCDC 2054147 and 2054148 contain the crystallographic data of compounds **1** and **2**, respectively.

### 4.3. X-ray Powder Diffraction

The X-ray powder diffractograms were collected for polycrystalline samples of compounds **2** and **3** filled into a 0.7 mm glass capillary that were mounted and aligned on a Empyrean PANalytical powder diffractometer (Malvern, UK), using CuKα radiation (λ = 1.54177 Å). A total of 3 scans were collected at room temperature in the range 5–40°. The experimental X-ray powder diffractogram of compounds **2** and **3** were compared with the simulated one from the X-ray single crystal structure of compounds **2** and XOYTUD, respectively, reported by Mercuri et al. [61].

### 4.4. Magnetic Measurements

DC Magnetic measurements were performed with a MPMS-XL-7 SQUID magnetometer (Quantum Design, San Diego, CA, USA) with an applied magnetic field of 1000 Oe (0.1 T) in the temperature range 2–300 K on polycrystalline samples of compounds **1**–**3** with masses of 2.961, 15.671 and 2.337 mg, respectively. Isothermal magnetization measurements were performed at 2 K with magnetic fields up to 7 T. AC susceptibility measurements were performed on the same samples with an oscillating magnetic field of 4 Oe at low temperatures in the frequency range 10–10,000 Hz with a Quantum Design PPMS-9 (Quantum Design, San Diego, CA, USA) and with different applied DC fields. Susceptibility data were corrected for the sample holders and for the diamagnetic contribution of the salts using Pascal’s constants [80].

### 4.5. Infrared Spectrospcopy

FT-IR spectra were performed on KBr pellets and collected with an Equinox 55 spectrometer (Bruker, Billerica, MA, USA). The spectra of compounds **1** and **2** and the band assignation are reported in the Supporting Information (Figure S17 and Table S2).

## 5. Conclusions

We have successfully synthesized and characterized two novel two-dimensional anilato-based compounds with single-ion and field-induced single-ion magnet behaviours. Compounds [Dy_2_(C_6_O_4_H_2_)_3_(dmso)_2_(H_2_O)_2_]·2dmso·18H_2_O (**1**) and [Dy_2_(C_6_O_4_Cl_2_)_3_(dmso)_4_]·2dmso·2H_2_O (**2**) present distorted hexagonal honeycomb lattices where each Dy(III) ion is connected to other three Dy(III) ions by bis-bidentate anilato bridges. Compound **1** is the first example of this kind of lattice (phase IIb) with dhbq^2−^ whereas compound **2** is the first example of phase IIb with Dy(III) and chloranilato.

Compounds **1** and **2**, as well as the related compound [Dy_2_(C_6_O_4_(CN)Cl)_3_(dmso)_6_] (**3**), reported by Mercuri et al. [61], show slow relaxation of the magnetization. Compound **1** shows SIM behaviour even when no DC field is applied that seems to relax following a quantum tunnelling mechanism. When a DC field is applied the relaxation of the magnetization in **1**–**3** follows Orbach and direct mechanisms with U_eff_ in the range 17.1–31.5 K.

In compounds **1**, **2** and **4**, we observe an almost linear dependence of the Ln τ_0_ and U_eff_ with the electronegativity of the X group of the anilato ligand and with the distortion of the TDD-8 coordination geometry of the Dy(III) ion.

The series of compounds here reported represents the second series prepared with Dy(III) and different anilato ligands and show that the use of anilato-type ligands with Dy(III) is an adequate strategy to prepare layered SIMs and FI-SIMs that may contain different coordinated and crystallization solvent molecules in the interlayer space and in the hexagonal channels that run perpendicular to the layers. The capacity, already demonstrated in similar systems [33], to evacuate and exchange these solvents opens the possibility to prepare two-dimensional MOFs with SIM behaviour and solvent exchange properties. Another interesting possibility offered by these compounds is the chemical or electrochemical reduction of some, or all, the anilato bridges in order to increase the magnetic coupling, as already observed in some anilato-bridged Dy(III) dimers [81,82], that may eventually lead to a 2D ferrimagnetic order.

## Figures and Tables

**Table 1:** Dy-O bond distances (in Å) for compounds **1** and **2**.

Atoms	1	2
Dy1-O2	2.319(11)	2.374(5)
Dy1-O3	2.378(13)	2.382(6)
Dy1-O12	2.437(12)	2.380(6)
Dy1-O13	2.388(12)	2.398(6)
Dy1-O22	2.348(13)	2.375(5)
Dy1-O23	2.325(13)	2.393(6)
Dy1-O1D	2.303(14)	2.319(6)
Dy1-O11W	2.357(14)	-
Dy1-O11D	-	2.293(6)
Dy1-O_anilato_^1^	2.365	2.384
Dy1-O_solvent_^2^	2.330	2.306

^1^ Average Dy-O_anilato_ distance; ^2^ Average Dy-O_solvent_ distance.

**Table 2:** Magnetic parameters for the relaxation processes in compounds **1**–**4**.

Compound	X	H (Oe)	Mechanisms	τ_QT_ (s)	τ_0_ (s)	U_eff_ (K)	AH^2^ (K^−1^)
**1**	H	0	QT	5.7(3) × 10^−6^	-	-	-
**1**	H	600	O + D	-	3.5(9) × 10^−8^	17.1(9)	5.8(2) × 10^4^
**2**	Cl	1000	O + D	-	6(1) × 10^−11^	31.5(7)	2.8(2) × 10^3^
**3**	Cl/CN	1000	O + D	-	4.6(5) × 10^−9^	21(3)	5.3(2) × 10^4^
**4**	Br	1000	O + D + QT	8.5(4) × 10^−7^	2.0(1) × 10^−9^	22.8(4)	1.0(3) × 10^5^

**Table 3:** X-ray crystal data collection for compounds [Dy_2_(C_6_O_4_H_2_)_3_(dmso)_2_(H_2_O)_2_]·2dmso·18H_2_O (**1**) and [Dy_2_(C_6_O_4_Cl_2_)_3_(dmso)_4_]·2dmso·2H_2_O (**2**).

Compound	1	2
CCDC	2054147	2054148
Formula	C_13_H_12_DyO_9_S_2_	C_15_H_14_O_10_DyCl_3_S_3_
*ρ_calc._* (g cm^−3^)	1.360	1.934
*μ* (mm^−1^)	3.026	3.648
Formula weight	538.85	719.29
Color	Dark Pink	Pink
Shape	Prism	Prism
Size	0.10 × 0.05 × 0.02	0.14 × 0.12 × 0.02
T (K)	119.6(8)	120(2)
Crystal System	monoclinic	Triclinic
Space group	*C*2*/c*	*P*-1
*a* (Å)	22.339(2)	9.7197(6)
*b* (Å)	16.2988(14)	10.6161(7)
*c* (Å)	14.5064(14)	12.7564(7)
α *(⁰)*	90	78.913(5)
β *(⁰)*	94.662(8)	73.305(5)
γ *(⁰)*	90	83.920(5)
V(Å^3^)	5264.3(8)	1235.46(14)
*Z*	8	2
Wavelength (Å)	0.71073	0.71073
Radiation type	MoKα	MoKα
θ_min_ (^o^)	6.04	3.632
θ_max_ (^o^)	50.1	24.070
Mesures Refl.	10245	8103
Independent Refl.	9970	4372
Reflections with I > 2σ(I)	7642	3680
*R_int_*	0.0633	0.0413
Parameters	238	301
Restraints	148	6
Largest pick (e^−^ Å^−3^)	7.30	1.652
Deepest Hole (e^−^ Å^−3^)	−4.26	−1.549
R_1_^a^	0.1184	0.0520
R_1_ (all data)	0.1396	0.0666
*w*R_2_^b^	0.2946	0.1127
*w*R_2_ (all data)	0.3164	0.1248
GooF ^c^	1.040	1.116

^a^ R_1_ = Ʃ||*F*_o_| − |*F*_c_||/Ʃ |*F*_o_|; ^b^ *w*R_2_ = [Ʃ*w*(*F*_o_^2^ − *F*_c_^2^)^2^ /Ʃ*w*(*F*_o_^2^)^2^]^1/2^; ^c^ GOF = [Ʃ[*w*(*F*_o_^2^ − *F*_c_^2^)^2^ /(N_obs_ − N_var_)]^1/2^.

## Data Availability

The data presented in this study are available on request from the corresponding author.

## Supplementary Materials

The following are available online, Figure S1: Thermal variation of the χ_m_T product and Isothermal magnetization at 2 K for compounds **1**–**3**, Figure S2: Frequency dependence of χ′_m_ for compound **1** with H_dc_ = 0 Oe and 600 Oe in the temperature range 1.9–4.0 K, Figure S3: Field dependence of χ″_m_ for compound **1** at different frequencies at 1.9 K, Figure S4: Frequency dependence of χ″_m_ for compound **1** at 1.9 K with different applied DC fields, Figure S5: Field dependence of the relaxation time, τ, for compound **1** at 1.9 K, Figure S6: Cole-Cole plot for compound **1** with H_dc_ = 0 Oe and 600 Oe, Figure S7: Field dependence of χ″_m_ for compound **2** at different frequencies at 1.9 K, Figure S8: Frequency dependence of χ″_m_ for compound **2** at 1.9 K with different applied DC fields, Figure S9: Field dependence of the relaxation time, τ, for compound **2** at 1.9 K, Figure S10: Cole-Cole plot for compound **2** with H_dc_ = 1000 Oe, Figure S11: Field dependence of χ″_m_ for compound **3** at different frequencies at 1.9 K, Figure S12: Cole-Cole plot for compound **3** with H_dc_ = 1000 Oe, Figure S13: Plot of the Ln τ_0_ vs. the distortion parameter from the ideal TDD-8 geometry and the Pauling electronegativity of the X group in compounds **1**, **2** and **4**, Figure S14: Plot of U_eff_ vs. the distortion parameter from the ideal TDD-8 geometry and the Pauling electronegativity of the X group in compounds **1**, **2** and **4**, Figure S15: X-ray powder diffractogram of compound **2** and the simulated one from the single crystal structure, Figure S16: X-ray powder diffractogram of compound **3** and the simulated one from the single crystal structure of compound XOYTUD, Figure S17: IR spectra in the 4000–400 cm^–1^ region of compounds **1** and **2**, Table S1: Continuous SHAPE measurement values of the 13 possible coordination geometries for the Dy(III) ion with coordination number eight in compounds **1** and **2** and Table S2: Selected vibrational frequencies (cm^−1^) for compounds **1** and **2**.
