# Supplementary material for: Slow Relaxation of the Magnetization in Anilato-Based Dy(III) 2D Lattices
Source: Molecules. 2021 Feb 23;26(4):1190. doi: 10.3390/molecules26041190 (PMC7926458; doi:10.3390/molecules26041190)
Supplement: Supplementary file 1 [file molecules-26-01190-s001.pdf]

Slow relaxation of the magnetization in anilato-based Dy(III) 2D lattices

Benmansour Samia *, Hernández-Paredes Antonio, Bayona-Andrés María and Gómez-García Carlos J. *

Dpt. Química Inorgánica. ICMol. Universidad de Valencia. C/ Catedrático José Beltrán, 2. 46980 Paterna (Valencia) Spain.

* Correspondence: sam.ben@uv.es (S.B.); carlos.gomez@uv.es (C.J.G.G.)

SUPPORTING INFORMATION

Continuous Shape Analysis

Table S1. Continuous SHAPE measurement (CShM)[1-3] values of the 13 possible coordination geometries for the Dy(III) ion with coordination number eight[4] in compounds **1** and **2**. The minimum values are indicated in bold.

Geometry	Symmetry	1	2
OP-8	D_{8h}	30.154	30.366
HPY-8	C_{7v}	22.635	23.156
HBPY-8	D_{6h}	15.212	15.039
CU-8	O_h	10.845	11.281
SAPR-8	D_{4d}	1.348	1.603
**TDD-8**	$\mathbf{D_{2d}}$	**0.902**	**1.208**
JGBF-8	D_{2d}	13.177	13.161
JETBPY-8	D_{3h}	29.264	28.647
JBTP-8	C_{2v}	2.502	2.267
BTPR-8	C_{2v}	1.908	1.806
JSD-8	D_{2d}	3.289	3.079
TT-8	T_d	11.667	12.011
ETBPY-8	D_{3h}	24.588	23.622

OP-8 = Octagon; HPY-8 = Heptagonal pyramid; HBPY-8 = Hexagonal bipyramid; CU-8 = Cube; SAPR-8 = Square antiprism; **TDD-8 = Triangular dodecahedron**; JGBF-8 = Johnson-Gyrobifastigium (J26); JETBPY-8 = Johnson-Elongated triangular bipyramid (J14); JBTP-8 = Johnson-Biaugmentedtrigonal prism (J50); BTPR-8 = Biaugmentedtrigonal prism; JSD-8 = Snub disphenoid (J84); TT-8 = Triakis tetrahedron.

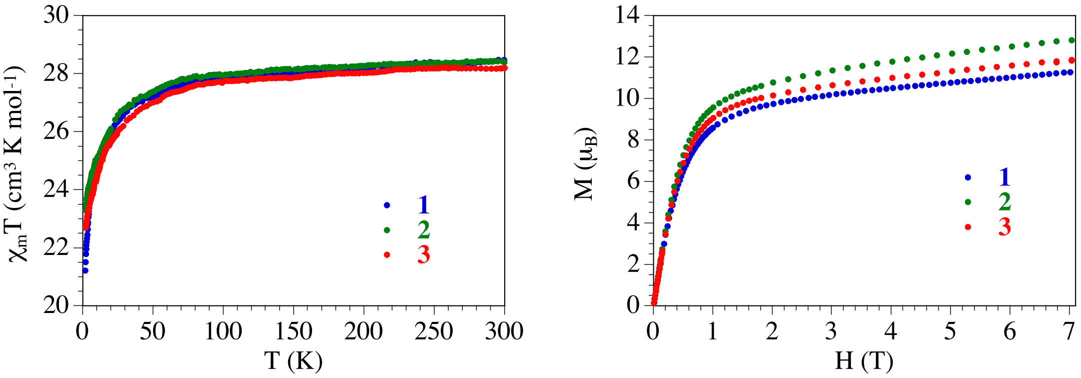

(**a**) (**b**)

Figure S1. (**a**) Thermal variation of the $\chi_m T$ product for compounds **1-3**; (**b**) Isothermal magnetization of compounds **1-3** at 2 K.

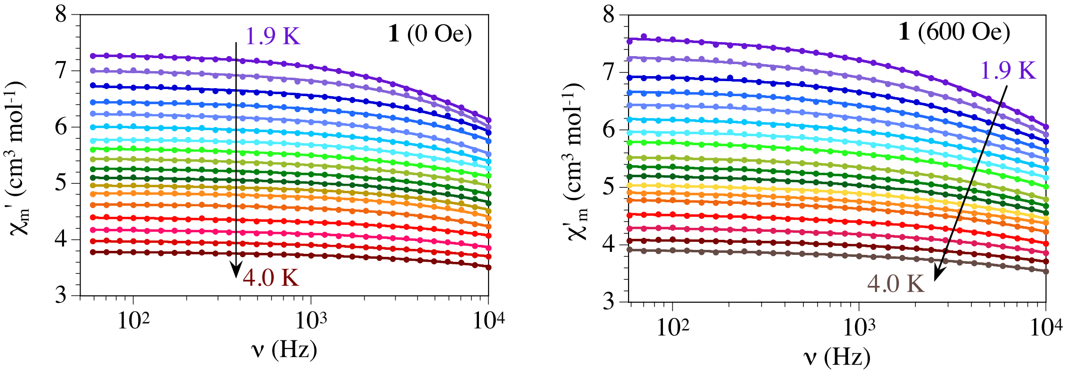

(**a**) (**b**)

Figure S2. Frequency dependence of χ'_m for compound **1** with H_{dc} = 0 Oe (**a**) and 600 Oe (**b**) in the temperature range 1.9-4.0 K. Solid lines are the best fit to the Debye model.

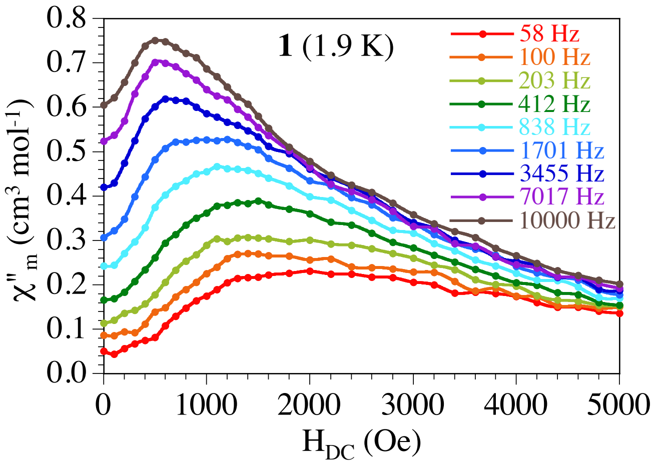

Figure S3. Field dependence of χ''_m for compound **1** at different frequencies at 1.9 K.

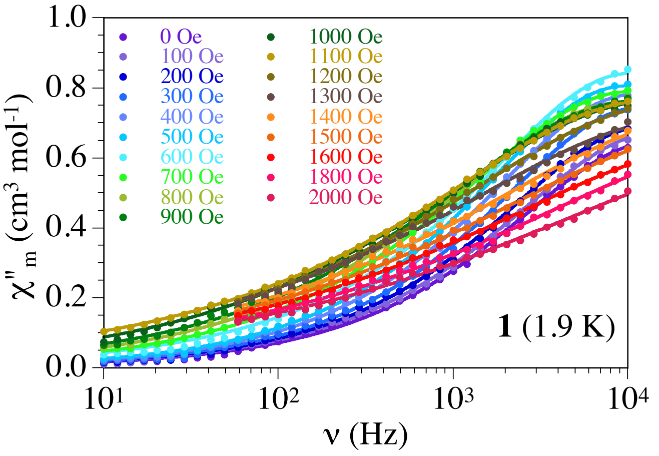

Figure S4. Frequency dependence of χ''_m for compound **1** at 1.9 K with different applied DC fields. Solid lines are the best fit to the Debye model.

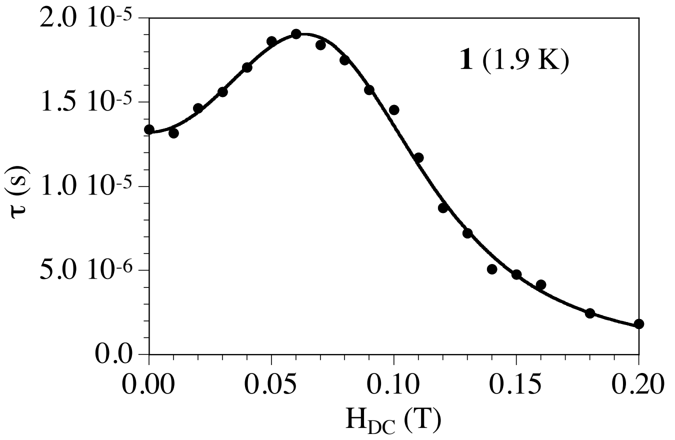

Figure S5. Field dependence of the relaxation time, τ, for compound **1** at 1.9 K. Solid line is the fit to the general model (equation 1 in the text) with: n = 4 (fixed value), A = 1.8(1)x10^{8} s^{-1} K^{-1} T^{-4}, B_1 = 4.8(4)x10^{4} s^{-1}, B_2 = 3.8(9)x10^{2} T^{-2} and D = 2.8(4)x10^{4} s^{-1}.

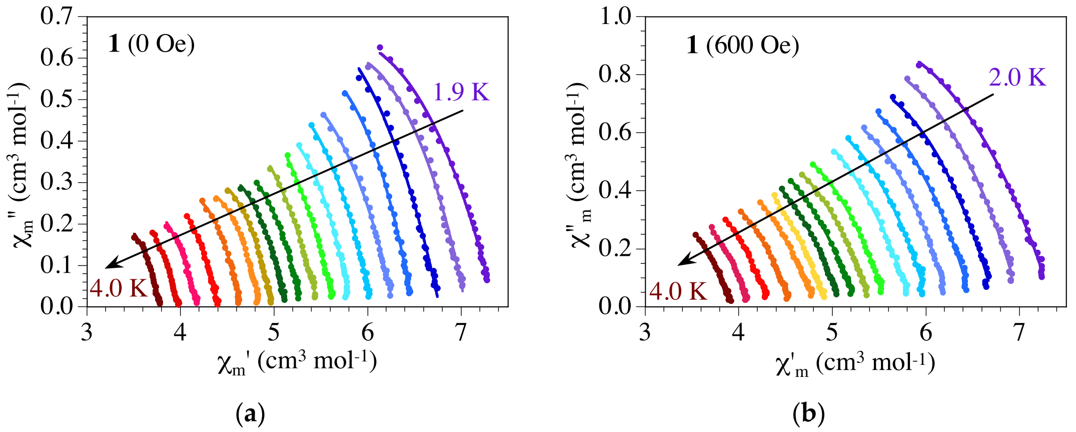

Figure S6. Cole-Cole plot for compound **1** with H_{dc} = 0 Oe (**a**) and 600 Oe (**b**). Solid lines are the best fit to the Debye model.

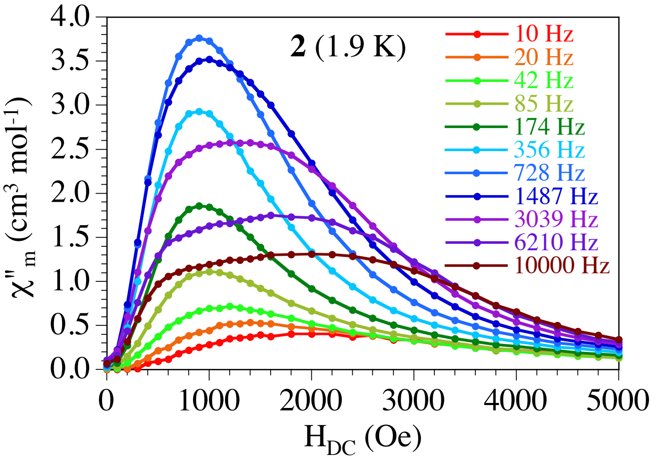

Figure S7. Field dependence of χ''_m for compound **2** at different frequencies at 1.9 K.

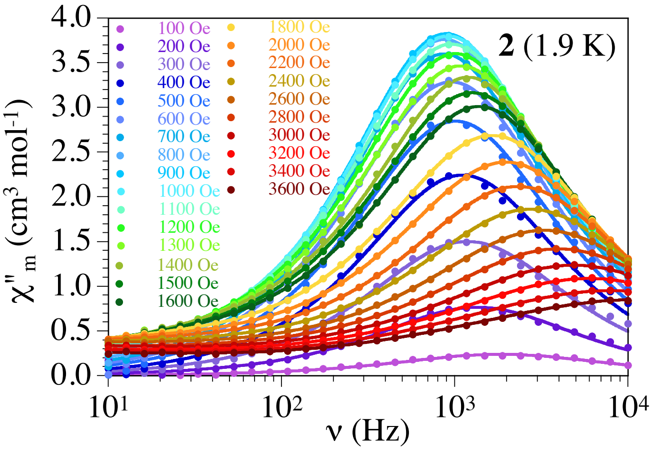

Figure S8. Frequency dependence of χ''_m for compound **2** at 1.9 K with different applied DC fields. Solid lines are the best fit to the Debye model.

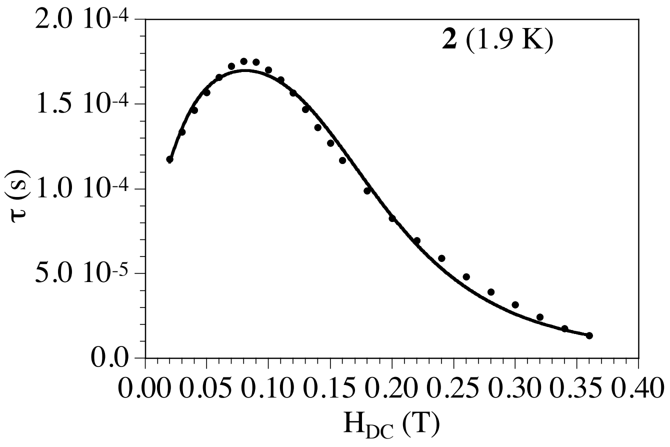

Figure S9. Field dependence of the relaxation time, τ, for compound **2** at 1.9 K. Solid line is the fit to the general model (equation 1 in the text) with n = 4 (fixed value), A = 2.1(1)x10^6 s^{-1} K^{-1} T^{-4}, B_1 = 6.6(1)x10^3 s^{-1}, B_2 = 2(1)x10^3 T^{-2} and D = 5.3(1)x10^3 s^{-1}.

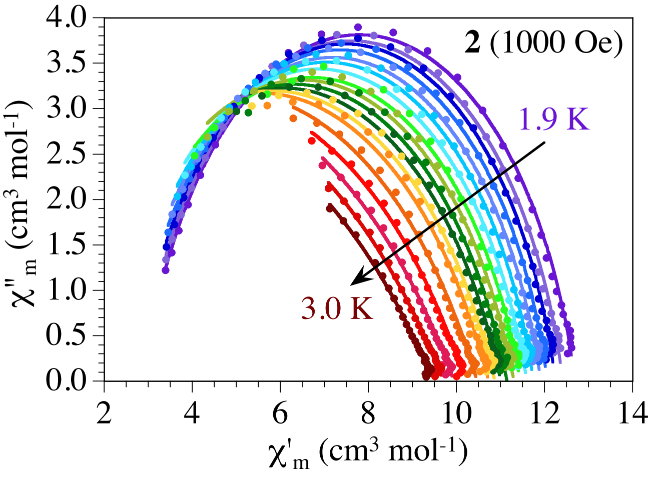

Figure S10. Cole-Cole plot for compound **2** with H_{dc} = 1000 Oe. Solid lines are the best fit to the Debye model.

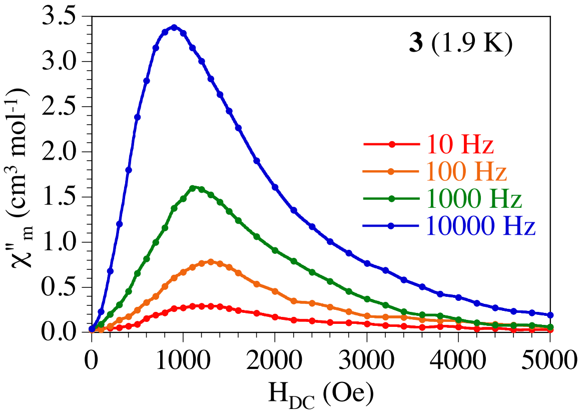

Figure S11. Field dependence of χ''_m for compound **3** at different frequencies at 1.9 K.

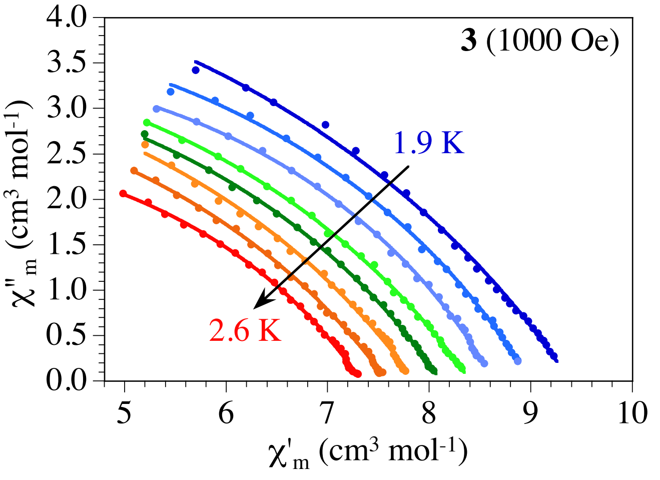

Figure S12. Cole-Cole plot for compound **3** with H_{dc} = 1000 Oe. Solid lines are the best fit to the Debye model with two relaxation processes.

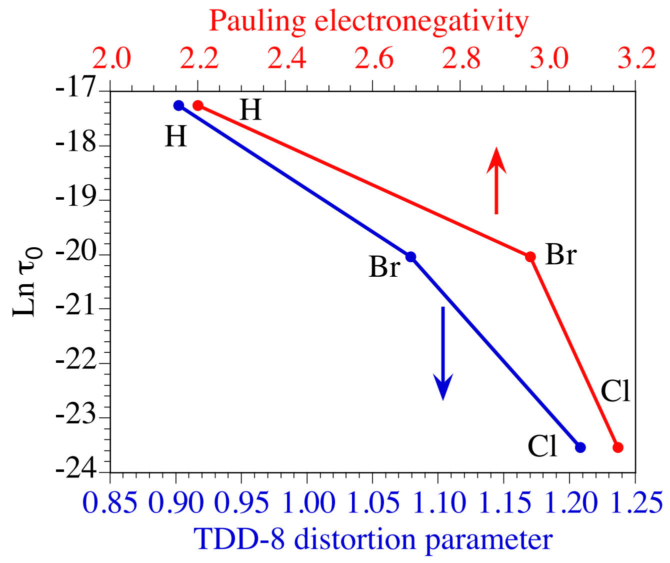

Figure S13. Plot of the Ln τ_0 *vs*. the distortion parameter from the ideal TDD-8 geometry (lower scale) and the Pauling electronegativity of the X group (upper scale) in compounds **1**, **2** and **4**.

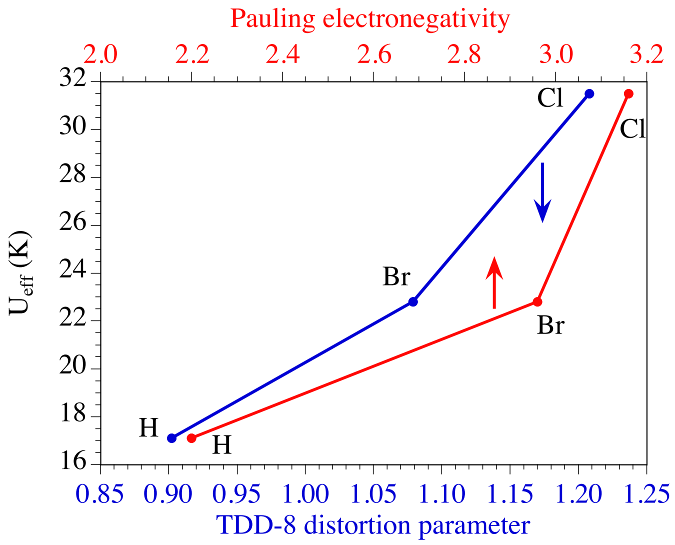

Figure S14. Plot of U_{eff} *vs*. the distortion parameter from the ideal TDD-8 geometry (lower scale) and the Pauling electronegativity of the X group (upper scale) in compounds **1**, **2** and **4**.

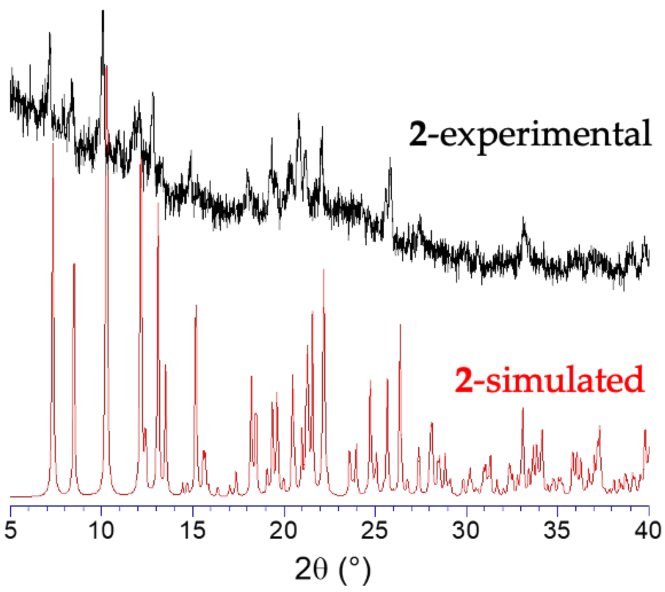

Figure S15. X-ray powder diffractogram of compound **2** and the simulated one from the single crystal structure.

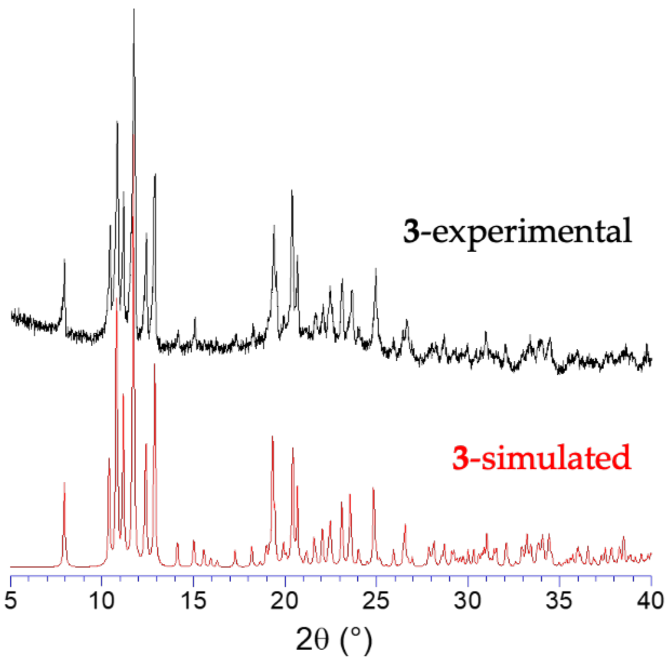

Figure S16. X-ray powder diffractogram of compound **3** and the simulated one from the single crystal structure of compound XOYTUD reported by Mercuri *et al*.[5]

Infrared spectroscopy (FT-IR)

Compounds **1** and **2** show very similar IR spectra (Figure S17) with differences attributed to the different anilato ligand in each compound: 2,5-dihydroxy-1,4-benzoquinone for **1** and chlorananilato for **2**. The main bands and their assignments are shown in Table S2.

Table S2. Selected vibrational frequencies (cm^{-1}) for compounds **1** and **2**.

Band	1	2
$\nu(C\text{-}H)_{solvent}$	2990	2998
	2910	2918
ν (C=C) + ν (C-O)	1522	1495
ν (C-C) + ν (C-O)	1374	1381
$\nu(S{=}O)_{dmso}$	1022	1000
$\rho(C\text{-}S)_{dmso}$	955	959
δ (C-Cl)	-	846
ρ (C-Cl)	-	579

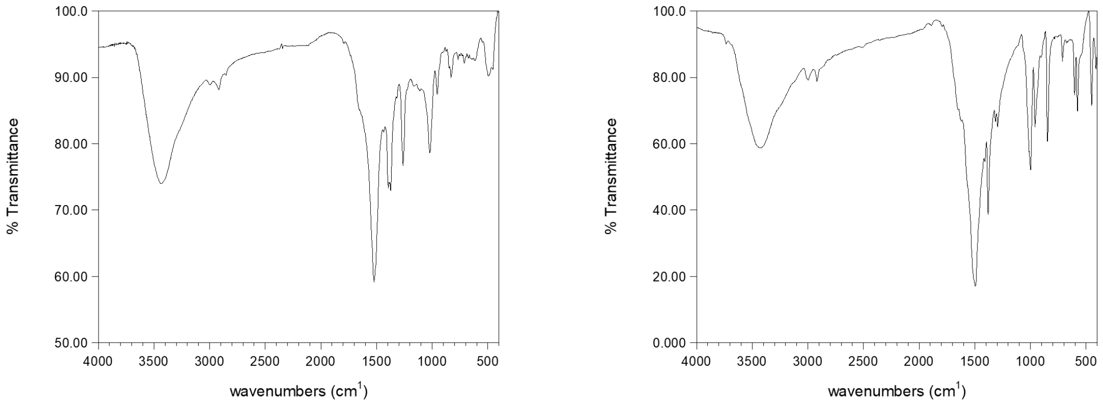

(**a**) (**b**)

Figure S17. IR spectra in the 4000-400 cm^{-1} region of compounds **1** (**a**) and **2** (**b**).

References

1 Llunell, M.; Casanova, D.; Cirera, J.; Bofill, J. M.; Alemany, P.; Álvarez, S.; Pinsky, M.; Avnir, D. Shape. **2013**, *2.3*.

2 Alvarez, S.; Alemany, P.; Casanova, D.; Cirera, J.; Llunell, M. Shape Maps and Polyhedral Interconversion Paths in Transition Metal Chemistry. *Coord. Chem. Rev.* **2005**, *249*, 1693-1708.

3 Álvarez, S. Distortion Pathways of Transition Metal Coordination Polyhedra Induced by Chelating Topology. *Chem. Rev.* **2015**, *115*, 13447-13483.

4 Casanova, D.; Llunell, M.; Alemany, P.; Alvarez, S. The Rich Stereochemistry of Eight-Vertex Polyhedra: A Continuous Shape Measures Study. *Chem. Eur. J.* **2005**, *11*, 1479-1494.

5 Sahadevan, S. A.; Monni, N.; Abhervé, A.; Cosquer, G.; Oggianu, M.; Ennas, G.; Yamashita, M.; Avarvari, N.; Mercuri, M. L. Dysprosium Chlorocyanoanilate-Based 2D-Layered Coordination Polymers. *Inorg. Chem.* **2019**, *58*, 13988-13998.
